# Supplementary material for: The causal role of circulating inflammatory markers in osteoporosis: a bidirectional Mendelian randomized study
Source: Front Immunol. 2024 Jul 18;15:1412298. doi: 10.3389/fimmu.2024.1412298 (PMC11291241; doi:10.3389/fimmu.2024.1412298)
Supplement: Supplementary file 1 [file DataSheet_1.zip › supplementary material/OSTEOPOROSIS-CIM/GCST90274814.tsv.gz_leave_one_out.pdf]

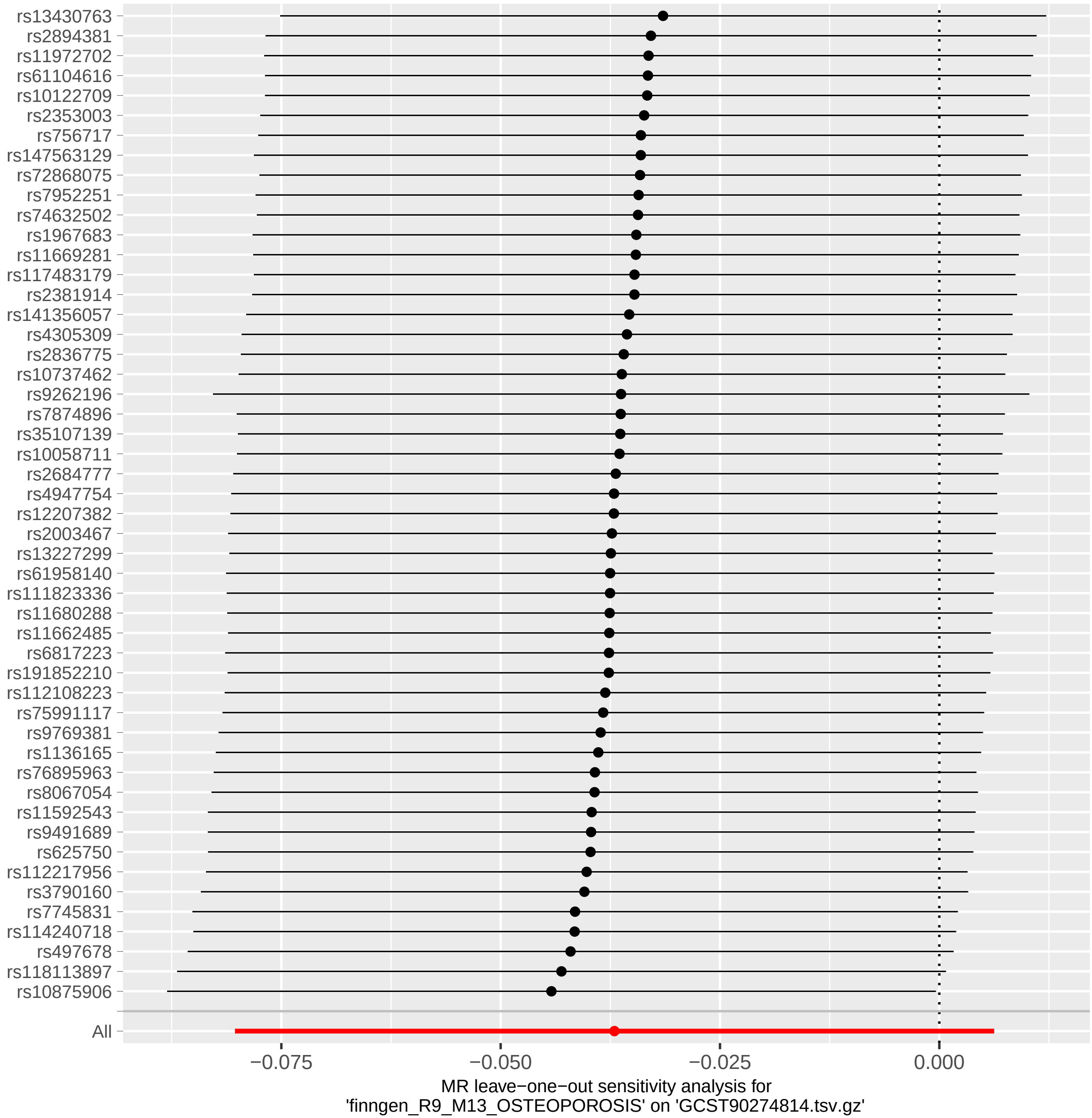
rs13430763
rs2894381
rs11972702
rs61104616
rs10122709
rs2353003
rs756717
rs147563129
rs72868075
rs7952251
rs74632502
rs1967683
rs11669281
rs117483179
rs2381914
rs141356057
rs4305309
rs2836775
rs10737462
rs9262196
rs7874896
rs35107139
rs10058711
rs2684777
rs4947754
rs12207382
rs2003467
rs13227299
rs61958140
rs111823336
rs11680288
rs11662485
rs6817223
rs191852210
rs112108223
rs75991117
rs9769381
rs1136165
rs76895963
rs8067054
rs11592543
rs9491689
rs625750
rs112217956
rs3790160
rs7745831
rs114240718
rs497678
rs118113897
rs10875906
All
−0.075
−0.050
−0.025
0.000
MR leave−one−out sensitivity analysis for
'finngen_R9_M13_OSTEOPOROSIS' on 'GCST90274814.tsv.gz'
